# Sphingosine-coating of plastic surfaces prevents ventilator-associated pneumonia

**DOI:** 10.1007/s00109-019-01800-1

**Published:** 2019-06-20

**Authors:** Aaron P. Seitz, Fabian Schumacher, Jennifer Baker, Matthias Soddemann, Barbara Wilker, Charles C. Caldwell, Ryan M. Gobble, Markus Kamler, Katrin Anne Becker, Sascha Beck, Burkhard Kleuser, Michael J. Edwards, Erich Gulbins

**Affiliations:** 1 Department of Surgery, College of Medicine, University of Cincinnati, 231 Albert Sabin Way ML 0558, Cincinnati, OH 45267 USA; 2 Institute of Nutritional Science, Department of Toxicology, University of Potsdam, Arthur-Scheunert-Allee 114-116, 14558 Nuthetal, Germany; 3 Department of Molecular Biology, University Hospital Essen, University of Duisburg-Essen, Hufelandstrasse 55, 45122 Essen, Germany; 4 Division of Research, Shriners Hospital for Children, Cincinnati, OH 45229 USA; 5 Thoracic Transplantation, University Hospital Essen, University of Duisburg-Essen, Hufelandstrasse 55, 45122 Essen, Germany; 6 Orthopedic Surgery, University Hospital Essen, University of Duisburg-Essen, Hufelandstrasse 55, 45122 Essen, Germany

**Keywords:** Coating, Plastic surfaces, Sphingosine, Ventilation, Acinetobacter baumannii, *Pseudomonas aeruginosa*, *Staphylococcus aureus*

## Abstract

**Abstract:**

Ventilator-associated pneumonia (VAP) is a major cause of morbidity and mortality in critically ill patients. Here, we employed the broad antibacterial effects of sphingosine to prevent VAP by developing a novel method of coating surfaces of endotracheal tubes with sphingosine and sphingosine analogs. Sphingosine and phytosphingosine coatings of endotracheal tubes prevent adherence and mediate killing of *Pseudomonas aeruginosa*, *Acinetobacter baumannii*, and *Staphylococcus aureus*, even in biofilms. Most importantly, sphingosine-coating of endotracheal tubes also prevented *P. aeruginosa* and *S. aureus* pneumonia in vivo. Coating of the tubes with sphingosine was stable, without obvious side effects on tracheal epithelial cells and did not induce inflammation. In summary, we describe a novel method to coat plastic surfaces and provide evidence for the application of sphingosine and phytosphingosine as novel antimicrobial coatings to prevent bacterial adherence and induce killing of pathogens on the surface of endotracheal tubes with potential to prevent biofilm formation and VAP.

**Key messages:**

- Novel dip-coating method to coat plastic surfaces with lipids.
- Sphingosine and phytosphingosine as novel antimicrobial coatings on plastic surface.
- Sphingosine coatings of endotracheal tubes prevent bacterial adherence and biofilms.
- Sphingosine coatings of endotracheal tubes induce killing of pathogens.
- Sphingosine coatings of endotracheal tubes ventilator-associated pneumonia.

## Introduction

Ventilator-associated pneumonia (VAP) is one of the most common nosocomial infections causing significant morbidity and mortality in critically ill patients. It affects up to 25% of mechanically ventilated patients and has an estimated mortality of 13% [1]. VAP adds 5–7 days to intensive care unit (ICU) length of stay and increases the length of hospitalization by 10–12 days [2].

Evidence-based practices, which have been identified and implemented to reduce rates of VAP, include semi-recumbent positioning, daily wake-wean trials, small bowel tube feeding, prophylactic probiotics, and early tracheostomy [1, 3, 4]. Evidence has shown silver-coated endotracheal tubes to be effective at reducing rates of VAP by 36% [1, 5]. However, silver-coated tubes are not seen in routine hospital use. A potential reason for this lack of implementation is the large upfront cost of implementing silver-coated endotracheal tubes compared to standard plastic tubes.

Bacteria responsible for VAP are diverse, however, 60% of causative agents are gram-negative bacteria, most commonly *P. aeruginosa* and *A. baumannii* [6]. The most common gram-positive bacterium involved in VAP is *S. aureus* [6]. The cause of VAP is multifactorial but is undeniably related to the presence of the endotracheal tube. Tracheal intubation inhibits the cough reflex, affects mucociliary clearance, provides direct access for bacteria from the upper to the lower respiratory system, and allows for the formation of biofilm, which can subsequently be a source of persistent infection [7]. Biofilms are increasingly becoming an area of focus because of their important roles in chronic or persistent infections often resistant to standard antibiotic therapy [8]. Bacterial adhesion is the first step in biofilm formation [9] and thus, prevention of bacterial adherence on the surface of endotracheal tubes could have potential to significantly reduce rates of VAP. In addition, killing of bacteria embedded in biofilms is a major clinical problem and development of drugs that kill pathogens in biofilms would be of great clinical interest.

Sphingolipids are a group of lipids that modulate multiple cellular functions. Sphingosine is a sphingoid long chain base that is generated from ceramide by ceramidases. Sphingosine has been previously shown to have antimicrobial properties against both gram-positive and gram-negative bacteria [10]. It has been described as an integral part of the innate immunity of the skin [11], oral mucosa [12], epithelial cells of the trachea and the bronchi [13–15], and the gingiva [16]. Sphingosine is expressed at high concentrations in epithelial cells of the trachea and large bronchi of the lung and mediates an immediate killing of pathogens such as *P. aeruginosa* or *S. aureus* in vivo [13–15]. Patients or mice with cystic fibrosis suffer from reduced concentrations of sphingosine in the airways resulting in their high infection susceptibility [15].

In the present study, we established a novel coating method to obtain high concentrations of sphingosine on plastic surfaces. Sphingosine-coating prevented adherence and growth of planktonic or biofilm *P. aeruginosa*, *S. aureus*, and *A. baumannii* on the tubes in vitro and in vivo as well as the development of pneumonia via the ventilation tube in a mouse model. Sphingosine coating was stable and nontoxic against tracheal epithelial cells.

## Results

### Sphingosine prevents bacterial adherence and growth on endotracheal tubes

**Fig. 1:**
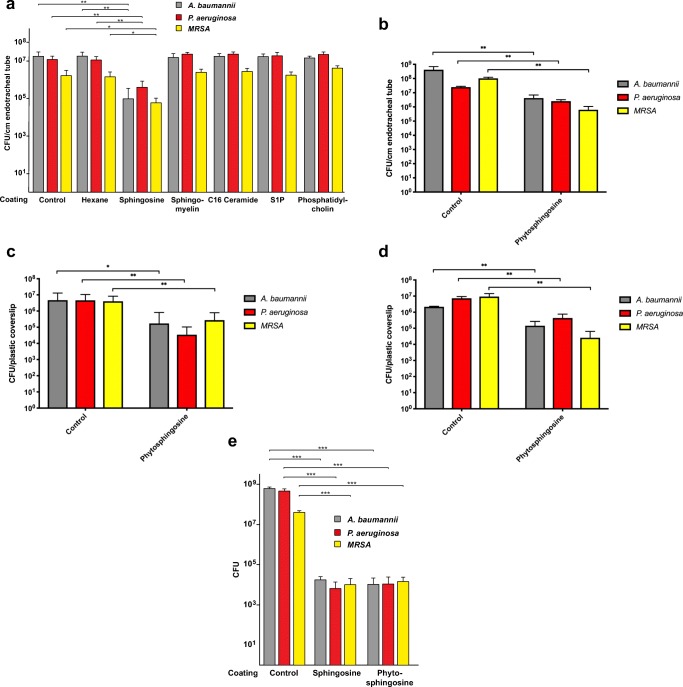
Antimicrobial efficacy of sphingosine and phytosphingosine-coated endotracheal tubes. **a** Uncoated vs vehicle (hexane)-coated vs sphingosine-coated and **b** vehicle (acetone)-coated vs phytosphingosine-coated 1-cm long segments of standard PVC endotracheal tubes were immersed for 12 h at 37 °C in bacterial suspension containing 1000 CFU of *A. baumannii*, *P. aeruginosa*, or methicillin-resistant *S. aureus* (*MRSA*). Tube segments were then rinsed in H/S, sonicated to release adherent bacteria and bacterial counts (colony forming units, CFU) were determined. Hexane-coating did not significantly reduce bacterial adherence and growth on the plastic surface. Sphingosine-coated segments prevented 99.4% (*p* < 0.005), 97% (p < 0.005), and 97% (*p* = 0.05) bacterial adherence of *A. baumannii* (*n* = 6), *P. aeruginosa* (*n* = 6), and *MRSA* (*n* = 3), respectively. Coating with sphingomyelin, C16 ceramide, sphingosine 1-phoshate, or phosphatidylcholine was without effect on bacterial adherence/growth (*n* = 6 each). **b** Phytosphingosine-coated segments prevented 99.0% (*p* = 0.009), 90% (*p* < 0.005), and 99.4% (*p* < 0.005) bacterial adherence of *A. baumannii* (*n* = 5), *P. aeruginosa* (*n* = 5), and *MRSA* (*n* = 5), respectively. **c** Bacterial suspension containing 10,000 CFU in growth media was pipetted onto plastic coverslips, covered with plastic film, and incubated for 24 h at 37 °C. Coverslips were rinsed with H/S, incubated for 12 h, adherent bacteria were released from the surface via sonication, plated and counted after overnight growth. Phytosphingosine-coated coverslips prevented 96% (*p* = 0.02), 99% (*p* = 0.006), and 93% (*p* < 0.005) bacterial adherence of *A. baumannii* (*n* = 20), *P. aeruginosa* (*n* = 15), and *MRSA* (*n* = 20), respectively. **d** Bacterial suspension containing 10,000 CFU in growth media was pipetted onto plastic coverslips, covered with plastic film, and incubated for 24 h. Additional suspensions of 10,000 CFU were pipetted onto the coverslips after 24 h and 48 h. At 72 h, CFU on the cover slips were determined. Phytosphingosine-coated coverslips prevented 93% (*p* = 0.005), 94% (*p* = 0.005), and 99% (*p* = 0.03) bacterial adherence and growth of *A. baumannii* (*n* = 3), *P. aeruginosa* (*n* = 3), and *MRSA* (*n* = 3), respectively. **e** Small parts of the tubes were coated as indicated, 10^4^ CFU of *P. aeruginosa*, *A. baumannii* or *S. aureus* were pipetted as a small drop onto the tube, incubated for 60 min, 500 μl TSB were added, and the bacteria were allowed to grow for 1 h. Aliquots of the cultures were then plated and CFU were determined after growth o/n (*n* = 6 each). Shown are mean ± SD, significant differences were compared to the respective control using ANOVA or *t* test, *p < 0.05, **p < 0.01, ***p < 0.001

Endotracheal tubes were coated with sphingosine and phytosphingosine in reagent grade hexane and acetone, respectively. Coating tube segments with solvent only did not significantly affect the adherence and growth of *P. aeruginosa*, *A. baumannii*, and methicillin-resistant *S. aureus* to the surface of the PVC compared to uncoated controls (*p* = 0.78, 0.63, 0.73, respectively), while sphingosine-coated and phytosphingosine-coated tube segments greatly reduced (more than 100-fold) adherence and growth of *P. aeruginosa*, *A. baumannii*, and methicillin-resistant *S. aureus* (MRSA) to the plastic surface (Fig. 1a, b). Coating of tubes with other lipids such as sphingomyelin, ceramide, sphingosine 1-phosphate, and phosphatidylcholine (all 30 mM) did not result in killing of the pathogens (Fig. 1a). Coating was controlled by addition of radioactive compounds and extraction of the lipids from the surfaces (not shown).

Quantifying bacteria adherent to surfaces after 24-h incubation immersed in bacterial suspension reaching greater than 10^7^ CFU/mL as in the experiments shown in Fig. 1 is not the most clinically relevant model of biofilm formation on endotracheal tubes. Endotracheal tubes in vivo are inoculated with bacteria, which adhere to the surface prior to biofilm formation. The source of the bacterial inoculant (i.e., oral secretions, gastric reflux, inhaled droplets, etc.) has been reviewed multiple times [17]. Regardless of the mechanism, bacteria adherent to the surface of an endotracheal tube are likely subjected to an environment exposed to humidified air. Thus, to simulate this condition, we modified our antimicrobial assay. Since bacteria adherent to endotracheal tubes were difficult to image via microscopy secondary to the curved nature of the tube, we coated flat plastic coverslips as a surrogate for PVC endotracheal tubes.

The antimicrobial assay we used is a variation of the international standard, ISO 22196 Test for Antimicrobial Activity of Plastics. Briefly, 10,000 CFU of *P. aeruginosa*, *A. baumannii*, and methicillin-resistant *S. aureus* in 10 μL TSB was placed on the coated coverslips, covered with a 2 cm × 3 cm piece of low-density polyethylene plastic film, incubated at 37 °C for 24 h and rinsed. In order to simulate the environment of an endotracheal tube in vivo, we then suspended the coverslips in air and incubated them at 37 °C, with 100% humidity for 12 h.

**Fig. 2:**
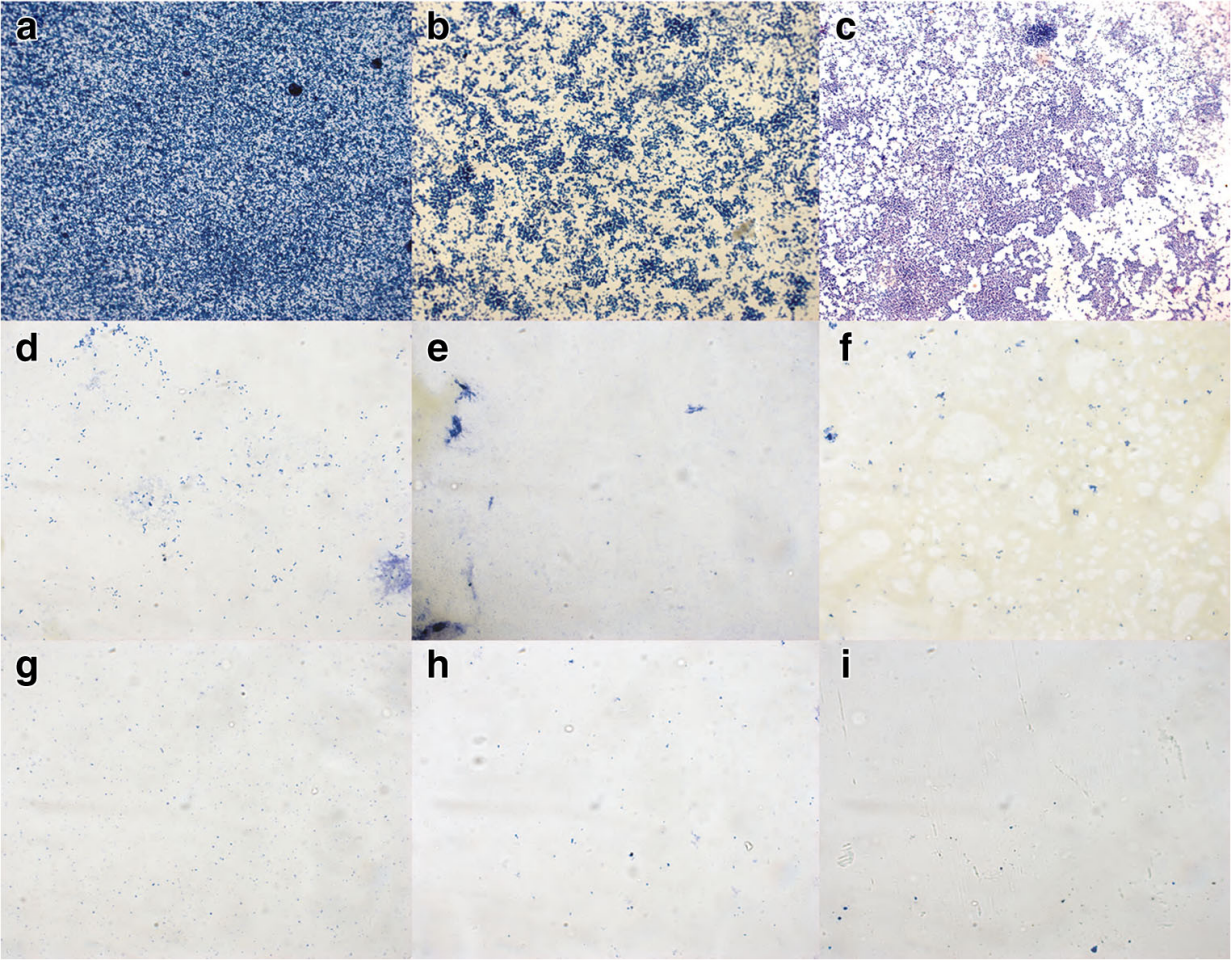
Antimicrobial efficacy of phytosphingosine and sphingosine-coated plastic. Displayed are the in vitro bacterial adherence and growth of, *P. aeruginosa* (**a, d, g**), methicillin-resistant *S. aureus* (*MRSA*) (**b, e, h**), and *A. baumannii* (**c, f, i**) to vehicle (ethanol)-treated (**a**, **b, c**) compared to sphingosine-coated (**d, e**, **f**), and phytosphingosine-coated (**g, h, i**) plastic coverslips. Plastic slides were fixed, stained with crystal violet, mounted on glass slides and bacteria were visualized by light microscopy (63 x). Shown are typical results from a total of three experiments

The results show that phytosphingosine reduced bacterial adherence and growth on the plastic surface by 93–99% compared to controls (Figs. 1c, d and 2). Similar results were obtained for sphingosine-coated surfaces.

In order to assess the durability of the sphingosine- and phytosphingosine-coating against bacteria, we performed the above protocol with the addition of a second and third inoculation of 10,000 CFU *P. aeruginosa*, *A. baumannii*, and methicillin-resistant *S. aureus* in 10 μL TSB at 24 h and 48 h. After 72 h, rinsing, dry incubation, and sonication were performed. As shown in Fig. 1d, we found the anti-adherent/anti-growth properties of phytosphingosine-coated plastic were durable at least up to 72 h. Similar results were obtained for sphingosine.

Finally, we tested whether coating of plastic tubes with sphingosine or phytosphingosine also results in killing of the bacteria. To this end, small parts of the tubes were coated with sphingosine or phytosphingosine or left uncoated or were treated with C_2_H_5_OH only, placed into wells of a 24-well plate and 10^4^ CFU of *P. aeruginosa*, *A. baumannii*, or *S. aureus* were pipetted as a small drop onto the tube. The samples were incubated for 60 min in a humidified atmosphere, 500 μl TSB was added and the remaining bacteria were allowed to grow for 1 h. Aliquots of the cultures were then plated. The results show that sphingosine or phytosphingosine coating killed *P. aeruginosa*, *A. baumannii*, or *S. aureus* on the surface of the plastic tubes. The data are presented in Fig. 1e.

### Sphingosine and phytosphingosine prevent biofilm colonization in vitro

**Fig. 3:**
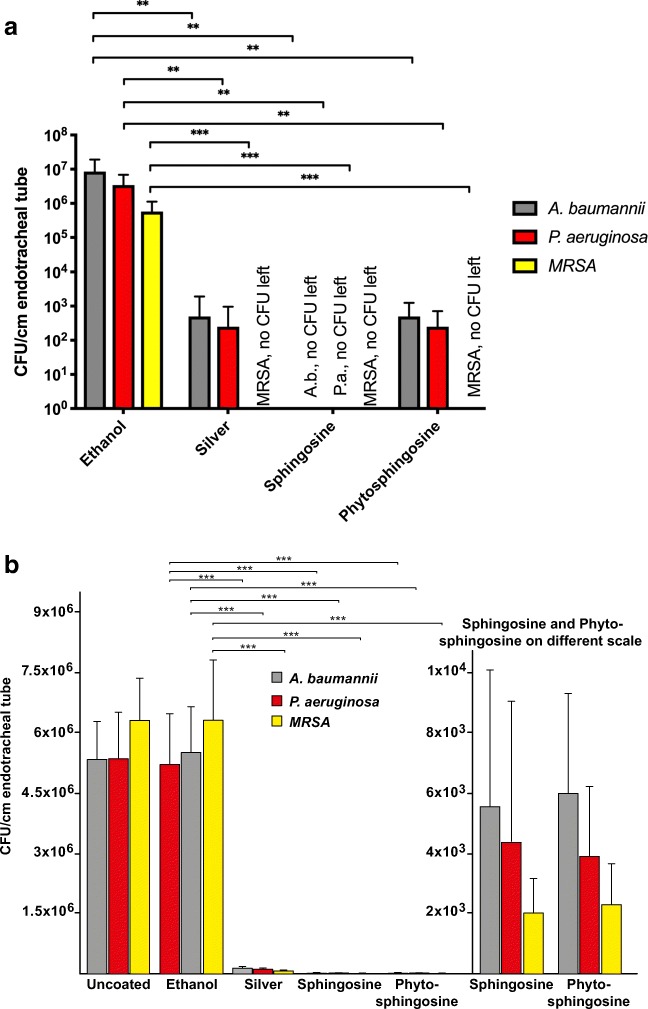
Antimicrobial efficacy of silver-coated, sphingosine-coated, and phytosphingosine-coated endotracheal tubes. **a** Endotracheal tube segments were challenged by placing 10 μL of bacterial suspensions in TSB onto the surface, incubated at 37 °C for 24 h, sonicated for 15 min to release adherent bacteria, and bacteria were plated and colonies were counted after overnight growth. Shown is the in vitro bacterial adherence and growth of *A. baumannii*, *P. aeruginosa*, and methicillin-resistant *S. aureus* (*MRSA*) to vehicle (ethanol)-treated, commercially available silver-coated, sphingosine-coated, and phytosphingosine-coated segments of 8.0 standard polyvinyl chloride endotracheal tubes. Silver-coated segments provided a 4.1, 3.8, and 6.5 log reduction against bacterial adherence of *P. aeruginosa*, *A. baumannii*, *and MRSA*, respectively, compared to ethanol-coated controls. Sphingosine-coated segments provided a 6.5 log reduction against all three strains and phytosphingosine-coated segments provided a 4.1, 3.8, and 6.5 log reduction against *P. aeruginosa*, *A. baumannii*, *and MRSA*, respectively, compared to ethanol-coated controls. Shown are mean ± SD, ANOVA, *p < 0.05, **p < 0.01, ***p < 0.001. **b***P. aeruginosa* strains 338, 345, and 762 were grown in biofilms, the biofilm containing the bacteria was removed from the plate and pipetted onto sphingosine-coated, phytosphingosine-coated, silver-coated, ethanol-only treated or uncoated/untreated plastic tubes, incubated for 60 min, bacteria were released, and the number of bacteria on the tube was determined by plating and overnight growth. Shown is the mean ± SD, *n* = 6 each, ANOVA, *p < 0.05, **p < 0.01, ***p < 0.001. The right panel shows the values for sphingosine and phytosphingosine on a different scale bar

The above results were obtained using a version of the international standard using PVC coverslips. This helped with imaging the biofilms, however, we also wanted to replicate in vivo condition with endotracheal tube segments. To more closely replicate the in vivo conditions, endotracheal tube segments were challenged with *P. aeruginosa*, *A. baumannii*, and methicillin-resistant *S. aureus* small volume suspensions pipetted directly on tube segments and incubated for 24 h. Adherent bacteria were released from the surface and quantified. The results of these tests showed that both, sphingosine and phytosphingosine, almost completely prevented adherence and growth of *P. aeruginosa*, *A. baumannii*, and methicillin-resistant *S. aureus* (Fig. 3a). Silver coating was effective against *P. aeruginosa* but failed to exhibit sufficient effects against *A. baumannii* and methicillin-resistant *S. aureus*.

In addition, we grew *P. aeruginosa* strains 338, 345, and PAO-1 as biofilms, removed the biofilm from the well plate, and added these preformed biofilms to sphingosine- or ethanol-coated plastic tubes. The samples were incubated for 60 min; tubes were washed; bacteria were released, plated, grown overnight; and colonies were counted. The results reveal that sphingosine-coating prevented the growth of bacteria within biofilms on the surface of plastic tubes (Fig. 3b).

In summary, these data indicate that plastic surfaces coated with sphingosine and phytosphingosine provide very high protection against infection with *P. aeruginosa*, *A. baumannii*, and MRSA, even under very stringent test conditions.

### Characterization of sphingosine and phytosphingosine-coated endotracheal tubes

**Fig. 4:**
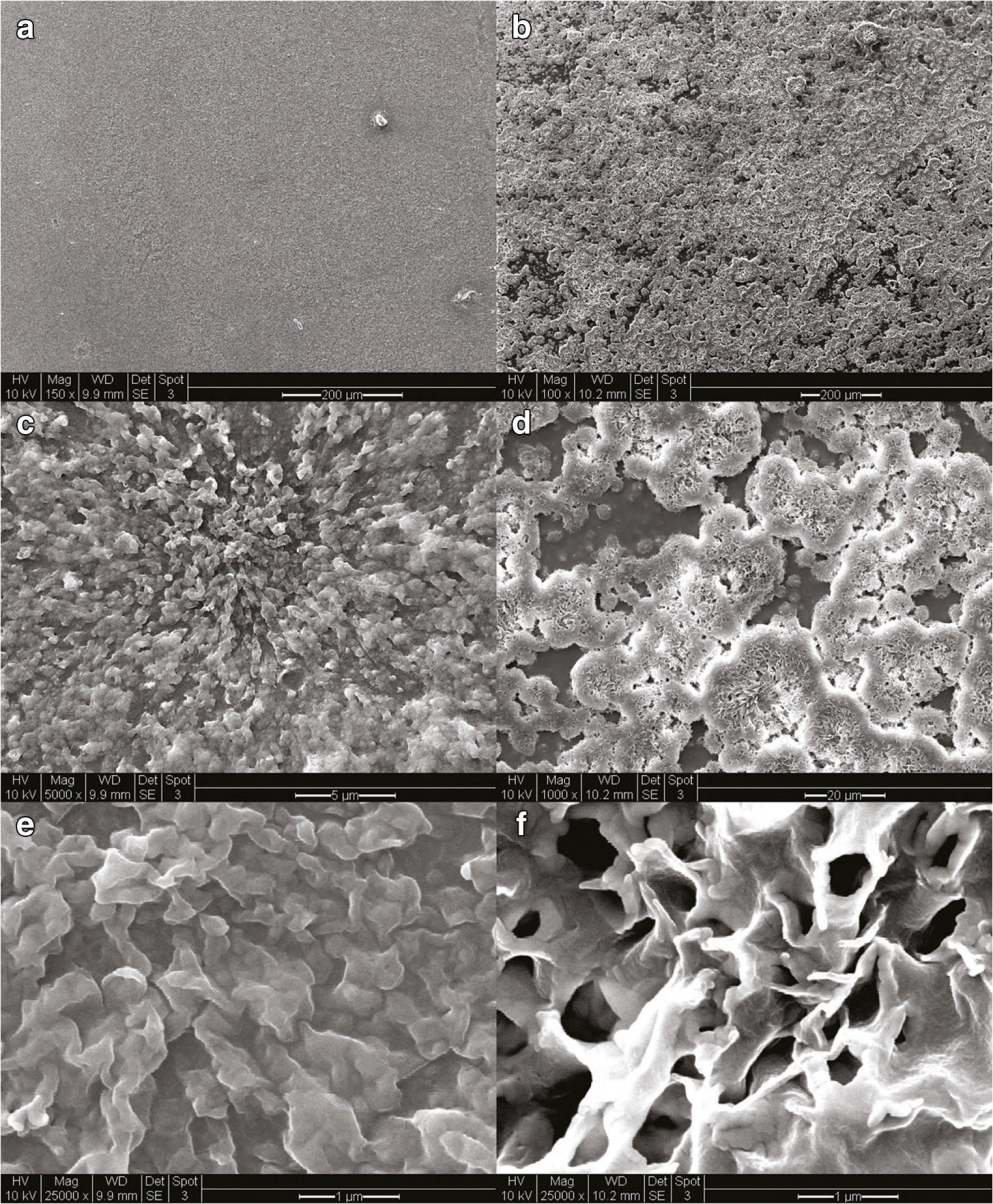

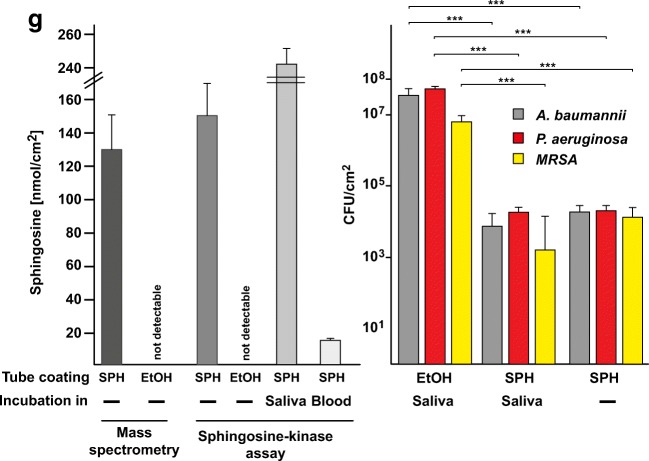
Morphology and quantity and durability of sphingosine/phytosphingosine coatings. 1 cm segments of endotracheal tubes were dip-coated once in **a**, **c**, **e** 30 mM sphingosine or **b**, **d**, **f** 30 mM phytosphingosine in 100% ethanol heated to 70 °C. Segments were then stained with 0.1% osmium tetroxide, sputter-coated with gold/platinum, and imaged with scanning electron microscopy. Images were obtained at different magnifications as indicated. Shown are representative figures from three independent studies. **g** Left panel: Endotracheal tube segments were dip coated in 30 mM sphingosine solution. Sphingosine was extracted and analyzed by mass spectrometry or measured by a sphingosine kinase assay on the tube. The sphingosine-coated endotracheal tube segments were also immersed in saliva or blood and incubated at 37 °C for 7 days prior to the sphingosine kinase assay. Right panel: Incubation of tube segments after the 7 day incubation in saliva with *P. aeruginosa*, *A. baumannii*, or *S. aureus* still kills the bacteria to a similar degree as without incubation of the coated tube with saliva. Experiments were performed as described in Fig. 1. Shown is the mean ± SD of the sphingosine concentration, *n* = 3 each or *n* = 6 for all infection experiments

Sphingosine and phytosphingosine are molecules found on various biological membranes of living organisms. They are also classified as nonionic biosurfactants. Adsorption of surfactants onto solid surfaces in aqueous solutions is a well-studied process. Multiple mathematical models have been developed to characterize this process (i.e., Langmuir isotherms) [18]. These models describe a process by which a monolayer (or bilayer) of surfactant molecules adsorb onto solid surfaces. Any attempt at increasing the aqueous concentration of the surfactant in order to increase the adsorption is limited by the critical micelle concentration of the surfactant [18]. Our method of molecular crystal thin film formation is not limited by the same parameters. As shown in Fig. 4a–f, our coating method results in adsorption of 3-dimensional surfactant structures with features as large as 20 μm in diameter. These three-dimensional structures, while not as organized as mono- or bilayer films, contain large amounts of adsorbed antimicrobial biosurfactant, i.e., sphingosine and phytosphingosine, while still < 10 μm in thickness.

### Quantification of sphingosine

To determine the total amount of sphingosine present on the surface of the endotracheal tubes after dip coating, we performed mass spectrometry (Fig. 4g) and a sphingosine kinase assay (Fig. 4g). Both methods confirmed a concentration of sphingosine in the nmol range per cm^2^. This is an impressive amount as sphingosine has been shown to kill bacteria in solution with concentrations of nmol/L to μmol/L [13].

### Durability of sphingosine coating

Any antimicrobial coating applied to endotracheal tubes (or any medical device) must have stability and durability when immersed in biological fluids. We studied the durability of sphingosine coating in blood and saliva for 7 days quantified by sphingosine kinase assay and in water, H/S, and PBS imaged with electron microscopy. As shown in Fig. 4g, sphingosine coating was stable in saliva, but not in blood. The amount of sphingosine quantified after soaking in saliva was nearly double of control. This indicates that sphingosine present in the saliva was adherent to the previously sphingosine-coated endotracheal tube pieces. Next, we tested whether a 7-day incubation of plastic intubation tubes with saliva affects killing/biofilm formation of with *P. aeruginosa*, *A. baumannii*, or *S. aureus* on the tube’s surface. To this end, plastic tubes were coated with sphingosine, incubated for 7 days in saliva, washed in H_2_O, and incubated with *P. aeruginosa*, *A. baumanni*, or *S. aureus*. As control, we used tubes that were also coated, but simply stored for 7 days. The results show that the coated tubes still killed the bacteria and incubation in saliva did not affect the potency of sphingosine coating to kill the bacteria (Fig. 4g).

Measuring the amount of sphingosine on the tubes also allowed us to test whether the sonication, which was used to release bacteria from the plastic surface, has an impact on killing. Sonication may release variable amounts of sphingosine into the buffer, which might be still active and kill released bacteria. To exclude killing of bacteria by released sphingosine, we neutralized sphingosine prior to sonication. Sphingosine binds to proteins, which neutralizes the anti-bacterial effects of sphingosine, and we therefore used a medium containing a high concentration of BSA (lipid-free; 100 g/L). In addition, we added sphingosine kinase 1 (10 μg with a specific activity of 2500 pmol/min/μg, i.e., 25 nmoles sphingosine/min are consumed), which converts sphingosine into sphingosine 1-phosphate, which does not kill bacteria (ref. 13 and Fig. 1a). We used 1 cm^2^ plastic in these experiments binding approximately 150 nmoles sphingosines (please see Fig. 4g). To ensure complete conversion of any sphingosine to sphingosine 1-phosphate and/or binding to BSA, we incubated the plastic pieces for 30 min with this solution, then sonicated and determined the bacterial numbers as above. The CFU obtained in these experiments were controls (no coating, no treatments): *P. aeruginosa*: (4.9 ± 0.90) × 10^6^ CFU; *A. baumannii*: (4.8 ± 0.88) × 10^6^ CFU; *S. aureus*: (4.2 ± 0.74) × 10^6^ CFU; coating with sphingosine + BSA and sphingosine kinase treatment: *P. aeruginosa*: (1.03 ± 0.94) × 10^3^ CFU; *A. baumannii*: (1.51 ± 1.02) × 10^3^ CFU; *S. aureus*: (1.83 ± 1.03) × 10^3^ CFU. The numbers did not significantly differ from those obtained without BSA and sphingosine kinase reported in Fig. 1c, indicating that killing of the bacteria/prevention of biofilm formation occurred prior to the release of the bacteria from the plastic.

Controls confirmed that addition of BSA plus sphingosine kinase to a solution of containing 150 nmoles sphingosine at a concentration of 150 μM sphingosine followed by inoculation with *P. aeruginosa*, *S. aureus*, and *A. baumannii* completely prevents killing of the bacteria, while a concentration of 150 μM sphingosine is sufficient to induce a 100% death of all bacteria (not shown).

The studies above establish a novel coating technique and demonstrate that coating of endotracheal tubes with sphingosine and phytosphingosine results in stable coatings of the plastic surfaces. The coated tubes prevent infection with *P. aeruginosa*, *A. baumannii* and methicillin-resistant *S. aureus*. However, these data are all in vitro. Therefore, we investigated whether coating of endotracheal/plastic tubes with sphingosine prevents bacterial pneumonia in vivo. We also determined the stability and potential toxicity of the coating in vivo.

### Sphingosine coating prevents *P. aeruginosa* and *S. aureus* pneumonia in vivo

**Fig. 5:**
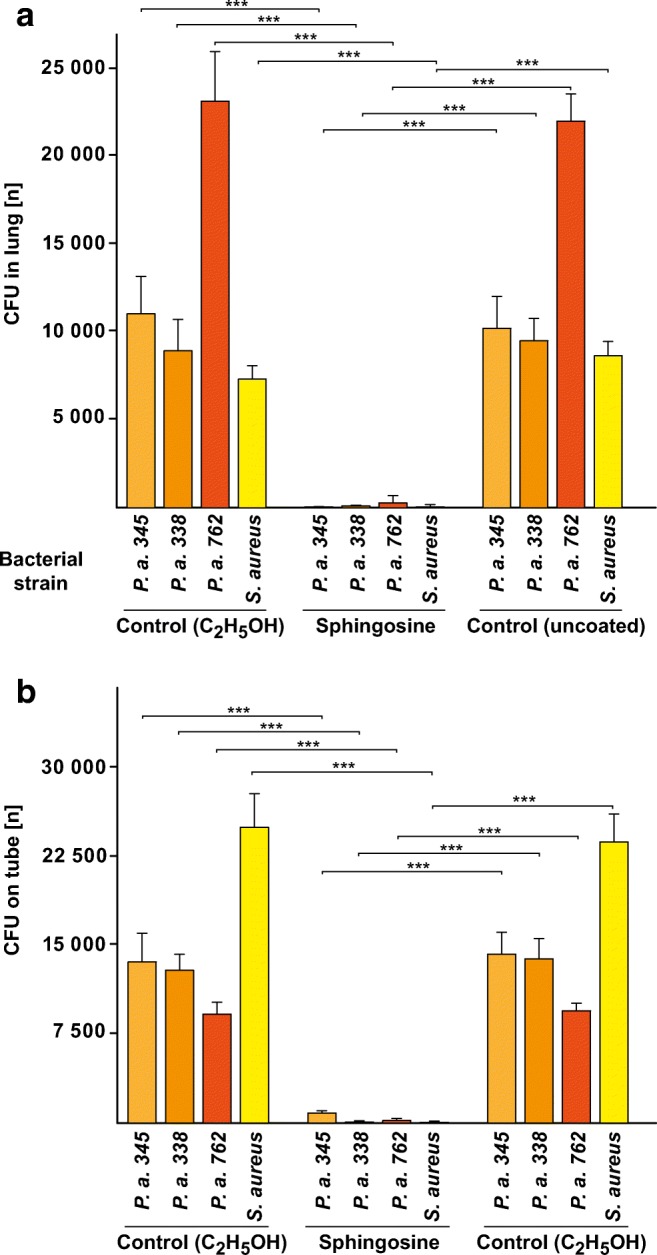
Sphingosine-coating of ventilation tubes prevents *P. aeruginosa* and *S. aureus* pneumonia. Mice were intubated, infected at the proximal outlet of the ventilation tube with 5 × 10^5^ CFU *P. aeruginosa* (*P. a.*) or a multi-resistant, clinical isolate of *S. aureus* in 2 μL H/S, the lungs were removed after 45 min and bacterial counts in the **a** lung and on the **b** tube were determined. Coating of the tubes with sphingosine prevented the infections. Mice were infected with three different *P. aeruginosa* strains, i.e., 338, 345, and 762, as well as a multi-resistant, clinical isolate of *S. aureus*. Ethanol-coating of the tube was without effect on bacterial infection. Shown is the mean ± SD of bacterial CFU in the **a** lung and the number of the bacteria on the **b** tube, *n* = 6 each, ANOVA, *** *p* < 0.001

VAP is a major clinical problem. To test whether coating of endotracheal tubes with sphingosine is able to prevent infections with *P. aeruginosa* and *S. aureus* via endotracheal tubes, we coated tubes with sphingosine in hexane or ethanol, or only with hexane or ethanol or left them uncoated. We used sphingosine in these experiments because it is normally present in eukaryotic cells and therefore will not result in an antigenic stimulation of lymphocytes. We then intubated mice into the trachea with these plastic tubes and infected the tube with 5 × 10^5^ CFU in 2 μl H/S applied at the outlet of the tube, approximately 5 mm proximal to the insertion site of the tube at the trachea. Mice were sacrificed 45 min after the infection; the lungs were removed, homogenized, and bacterial CFU were determined after overnight growth on LB plates. Mice intubated with uncoated or solvent only coated tubes developed severe pneumonia (Fig. 5a, b), while mice intubated with sphingosine-coated tubes were protected. Sphingosine coating completely protected the mice from pneumonia after infection with the *P. aeruginosa* strains 338 and 345 and a multi-resistant, clinical *S. aureus* isolate, and reduced the bacterial CFU in the lung or on the tubes by more than 98%, even after infection with the very aggressive and highly motile clinical *P. aeruginosa* strain 762 (19) (Fig. 5a, b). No difference was noted between sphingosine-coating with hexane or ethanol as solvent (not shown).

### Sphingosine coating of endotracheal tubes is stable in vivo

**Fig. 6:**
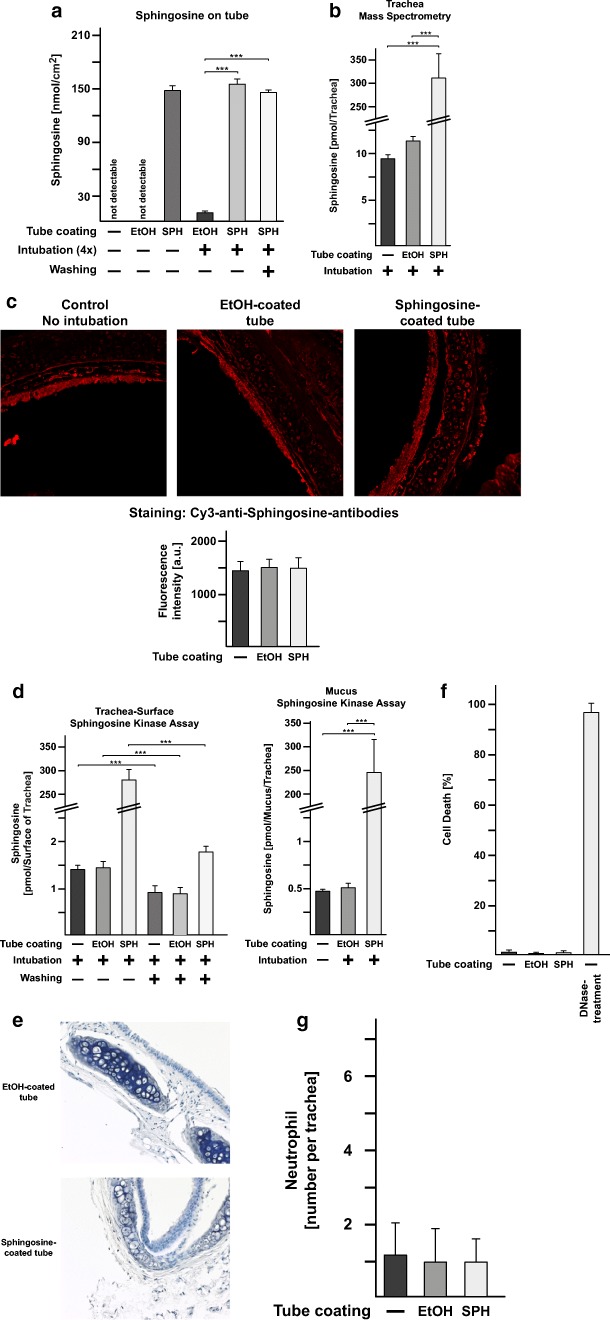
Sphingosine coating of endotracheal tubes is stable in vivo, non-toxic to tracheal epithelial cells, and does not induce inflammation. **a** Tubes were sphingosine (SPH) or ethanol (EtOH)-coated and inserted over a total of 4 h into mice. To avoid variations due to prolonged and very deep anesthesia, we intubated 4 mice consecutively on 4 days for 1 h each. The concentration of sphingosine on the tube was determined by a sphingosine-kinase assay. Sphingosine concentrations on the tubes remained stable and even slightly increased, very likely by binding sphingosine-containing mucus, which is removed by washing the tube. Likewise, ethanol-coated tubes contained low amounts of sphingosine on the surface after intubation. Ethanol-coated or uncoated tubes (not used for intubation) were negative. Shown are the mean ± SD of sphingosine concentrations in pmol/cm^2^, *n* = 6 each, ANOVA, *p < 0.05, **p < 0.01, ***p < 0.001. **b** Mass spectrometry reveals that intubation of sphingosine-coated tubes results in a marked increase of total sphingosine in the trachea. Mice were intubated as above for 60 min, the tube removed, the trachea removed and subjected to mass spectrometry. Shown is the mean of the concentration of sphingosine ± SD, *n* = 3–5 each, ANOVA, *p < 0.05, **p < 0.01, ***p < 0.001. **c** Immunofluorescence microscopy analysis of paraffin sections of trachea stained with Cy3-coupled anti-sphingosine antibodies does not reveal an increase of sphingosine in the epithelial cells of the trachea or the submucosa or any other cell of the trachea. Shown are typical results and the mean ± SD of the quantification of the fluorescence staining of each 100 cells of the trachea from 6 independent samples per group. The fluorescence intensity is given in arbitrary units. **d** In situ surface sphingosine-kinase assays on trachea from intubated mice reveal that most of the sphingosine released from the tube remains in the mucus of the trachea. Washing the trachea prior to the kinase assay removes the increase of sphingosine detected after intubation, which is recovered in the mucus in the wash buffer. Shown is the mean of the concentration of sphingosine ± SD per trachea, *n* = 6 each, ANOVA, *p < 0.05, **p < 0.01, ***p < 0.001. To exclude toxic or pro-inflammatory effects of sphingosine-coated tubes, we performed hemalaun, Cy3-coupled anti-Gr1-antibody and TUNEL stainings of paraffin sections of the trachea from mice that were intubated with sphingosine- or ethanol (EtOH)-coated ventilation tubes. We did not observe any **e** structural damage, **f** induction of cell death, or **g** influx of granulocytes/monocytes into the submucosa epithelial cell layer in the trachea of mice intubated with sphingosine-coated plastic tubes. Displayed are typical examples of the stainings and the quantitative analysis of the number of dead epithelial cells and the influx of leukocytes, which were quantified in 10 sections from each trachea from 6 mice, i.e., a total of 60 sections. In each section, the entire epithelial cell layer and the submucosa of the trachea were investigated. Structural damage was determined as the number of disruptions of the epithelial cell layer. Shown are the mean ± SD, *n* = 6 each, ANOVA, *p < 0.05, **p < 0.01, ***p < 0.001

To test the stability of sphingosine after coating on endotracheal tubes in vivo, we inserted a tube over a total of 4 h into mice. To avoid variations due to prolonged and very deep anesthesia, we intubated 4 mice consecutively on 4 days for 1 h each. We then determined the concentration of sphingosine on the outer and inner surface of the tube by a sphingosine-kinase assay. Ethanol-only coated and uncoated tubes that were also employed for intubation and sphingosine-coated tubes that were not used for intubation served as controls. To discriminate between sphingosine that is directly bound to the plastic and sphingosine that might have been released and binds within mucus that sticks to the tube, we measured sphingosine on the tubes with or without washing in H/S. The results show that the concentration of sphingosine on tubes did not significantly decrease with intubation (Fig. 6a). Washing the tubes in H/S slightly reduced the sphingosine amount on the tube. The ethanol-only coated tubes also contained some sphingosine on the surface after intubation, but much less than the sphingosine-coated tubes (Fig. 6a). Thus, the tubes very likely bind mucus and thereby sphingosine is present in the mucus of the airways. Very similar results were obtained for coating tubes in sphingosine in hexane or hexane as control (not shown).

### Sphingosine released from the intubation tube accumulates in the mucus of the trachea

Next, we investigated how much sphingosine is transferred from the tube into the trachea and, in particular, onto the surface of the tracheal epithelial cells. To this end, we intubated the mice for 60 min with sphingosine or ethanol-only coated tubes, removed the tube, and determined the concentration of sphingosine in the total trachea by mass spectrometry. These experiments revealed a marked increase of sphingosine in the trachea compared to the levels in mice intubated with control tubes (Fig. 6b), although this amount is still only a very minor fraction of the tube-bound sphingosine. To determine the topology of sphingosine accumulation after intubation with sphingosine-coated tubes, we performed stainings of trachea from mice that were intubated with sphingosine- or control-coated tubes for 60 min, with anti-sphingosine antibodies. Surprisingly, the results did not reveal a significant accumulation of sphingosine in tracheal epithelial or any other cell of the trachea (Fig. 6c). This finding suggests that sphingosine released from the tubes accumulates in the mucus on top of the epithelial cells. This mucus is washed off during the fixation of the trachea and, thus, any sphingosine within the mucus would be removed. To test this hypothesis, we performed in situ surface sphingosine kinase assay and determined the concentration of sphingosine on the surface of trachea that was subjected to a surface kinase assay after intubation and removal of the trachea followed by extensive washing in H/S prior to the kinase assay or immediate kinase assay without any washing. In addition, we also measured the amount of sphingosine in the wash solution. The results reveal an increase of sphingosine on the surface of unwashed trachea, immediately subjected to surface sphingosine kinase assays after intubation (Fig. 6d). Washing of the trachea abrogated the increase of sphingosine on the surface of the trachea after intubation with sphingosine-coated tubes (Fig. 6d). Sphingosine was recovered in the wash solution (Fig. 6d). This indicates that sphingosine released from the surface of the intubation tube remained in the mucus on the tracheal epithelial cells. The total amount of sphingosine on the tube inserted in the trachea was 29.1 ± 1.8 nmol (Fig. 6a shows values normalized to 1 cm^2^, the surface of the inserted tube is approximately 0.25 cm^2^). Thus, approximately only 1% (~ 300 pmol) of the total sphingosine bound to the tube was released into the mucus.

### Sphingosine coating of endotracheal tubes is not toxic or pro-inflammatory in vivo

To exclude toxic or pro-inflammatory effects of sphingosine-coated tubes, we intubated mice for 2 h with sphingosine-coated tubes or the appropriate controls, removed the trachea, fixed the trachea in PFA, and performed H&E, Cy3-coupled anti-Gr1-antibody, and TUNEL stainings of paraffin sections. We did not observe any structural damage, influx of granulocytes/monocytes, or induction of cell death in the trachea of mice intubated with sphingosine-coated plastic tubes (Fig. 6e–g).

## Discussion

Our data show that sphingosine- and phytosphingosine-coated endotracheal tubes are highly efficacious at preventing bacterial adherence and growth against three of the most common pathogens, *P. aeruginosa*, *A. baumannii*, and *S. aureus*, associated with VAP when compared with standard plasticized PVC endotracheal tubes under a variety of conditions. Additionally, we have shown that the sphingolipid molecular crystal thin film coating is effective, stable, and nontoxic in vivo. Although only a small portion of coated sphingosine is released from the tubes, the absolute amount of sphingosine is approximately 300 pmoles. However, our studies demonstrate that most of the released sphingosine remains in the mucus on top of the epithelial cells of the trachea and only a small portion is incorporated into the epithelial cells. In accordance, tracheal epithelial cells from intubated mice do not show any signs of cell death and no influx of leukocytes into the trachea mucosa was observed, consistent with findings of our group that repeated inhalations of high concentrations of sphingosine, i.e., up to 1 mM on 10 separate treatments over 11 days, had no evidence of toxicity [20]. The observation that some sphingosine is slowly released from the surface of the tubes into the mucus also implies that such an intubation may even improve the bactericidal effects of airway mucus and, thus, contribute to the protection from a VAP.

VAP continues to be a major cause of morbidity and mortality in critically ill patients. While prompt diagnosis and effective treatment with standard antibiotic regimens are important in mitigating the detrimental effects of VAP, development and implementation of more effective prevention strategies will decrease the incidence and likely provide a greater reduction in morbidity and mortality. Semi-recumbent positioning, chlorhexidine oral care, and subglottic suctioning have all been shown to reduce rates of VAP [21]. Silver-coated endotracheal tubes have also been shown to reduce rates of VAP [5, 22], but have not received widespread implementation. Silver coating provides clear protection against bacteria, however, coating with sphingosine provides similar or better protection against *P. aeruginosa*, *S. aureus*, and *A. baumannii* and even improves the natural defense systems of the mucociliary system in the upper airways.

The cause of VAP is multifactorial, but the presence of a biofilm that develops after only 24 h of tracheal intubation has been identified as a likely source of infection [23, 24]. The coating of plastic with sphingosine provides the advantage to prevent adherence of the bacteria and to kill the pathogens and, thus, has the potential to affect meaningful change in the prevention of VAP. Our in vivo experiments are only done for a total of 4 h because it is very difficult to perform long-term intubation experiments on mice. However, since the bacteria are killed already within the 45-min intubation period, we assume that a similarly fast killing occurs in longer time courses. In addition, sphingosine-coating on the tubes could be periodically restored by applying sphingosine by ventilation of sphingosine via the tube. Thus, sphingosine-coated tubes are very likely and also suitable in longer time courses.

We have discovered an interesting novel method that could be applied to other bioactive molecules and other surfaces in the formation of molecular crystal films. Our coating process utilizes an evaporation-induced molecular crystallization process that leaves a thin film of SPH.

At present, it is unknown how sphingosine kills bacteria. It is known that micellar sphingosine kills many pathogens including *E. coli*, *P. aeruginosa*, *S. aureus*, *A. baumannii*, *Moraxella catarrhalis*, *Haemophilus influenzae*, *Burkholderia cepacia*, *Neisseria meningitides*, and *Neisseria gonorrhoeae* [10–15, 25]. It is possible that sphingosine simply kills pathogens by its biophysical properties, which would also suggest that sphingosine’s antimicrobial mechanism is not prone to development of bacterial resistance. On the other hand, bacteria express sphingosine-responsive elements [26], suggesting that sphingosine may also have some biochemical effects in bacteria.

In summary, sphingosine and phytosphingosine-coating effectively prevent adherence of *P. aeruginosa*, *S. aureus*, and *A. baumannii* to the surface of endotracheal tubes with an effectiveness comparable to silver-coating. Sphingosine coating on endotracheal tubes prevents *P. aeruginosa* and *S. aureus* infection over endotracheal tubes, is stable over time, and is safe in vivo. In vitro, sphingosine and phytosphingosine were shown to have antimicrobial durability of at least 4 days and a coating durability in aqueous solutions and saliva of at least 7 days. Future large animal studies are necessary to establish the safety of sphingolipid coatings and future randomized clinical trials will be necessary to determine sphingosine’s or phytosphingosine’s ability to provide a cost-effective preventative strategy to reduce rates of VAP.

## Conclusions

We describe a novel method to coat plastic surfaces and provide evidence for the application of sphingosine and phytosphingosine as novel antimicrobial coatings to prevent bacterial adherence and induce killing of pathogens on the surface of endotracheal tubes with potential to prevent biofilm formation and ventilator-associated pneumonia.

## Materials and methods

### Animal procedures

The study protocol was reviewed and approved by the University of Cincinnati Institutional Animal Care and Use Committee (IACUC) and USAF Surgeon General Office of Research Oversight & Compliance. Animals were handled and studies were conducted under a program of animal care accredited by the Association for Assessment and Accreditation of Laboratory Animal Care International (AAALAC) and in accordance with the “Guide for the Care and Use of Laboratory Animals” (NRC, 2011; in compliance with DoDI 3216.1).

### Materials

D-erythrosphingosine (d18:1), D-ribo-phytosphingosine, C16 ceramide, sphingomyelin, and sphingosine 1-phosphate (S1P) were purchased from Avanti Polar Lipids (Alabaster, AL), phosphatidylcholine from Sigma (Deisenhofen, Germany). Anhydrous hexane and acetone were purchased from Sigma-Aldrich (St. Louis, MO). [^3^H]- or [^14^C]-labeled sphingomyelin, C16 ceramide, and phosphatidylcholine were from Perkin Elmer, [^3^H] S1P from ARC. BSA was from Sigma, Sphingosine kinase 1 form R&D. Absolute, molecular biology grade ethanol was purchased from Fisher Scientific (Pittsburgh, PA). Plasticized polyvinyl chloride (PVC) endotracheal tubes (8.0 mm) were purchased from Cardinal Health (Dublin, OH). Silver-coated endotracheal tubes (8.0 mm) were from Bard Medical (Covington, GA). PVC coverslips (24 × 60 mm) were purchased from Electron Microscopy Sciences (Hatfield, PA). Small PVC vein catheters (21 gauge; Braun Melsungen, Melsungen, Germany) were used in the mouse experiments. Three different bacterial species were used: Two strains of *S. aureus*, i.e., the methicillin-resistant strain USA 300 and a multi-resistant clinical isolate [27], a clinical isolate of *A. baumannii* and 4 different *P. aeruginosa* strains, i.e., three clinical strains named 338, 345, and 762 [19], as well as the laboratory strain PAO-1. The *P. aeruginosa* strains 338 and 345 were mucoid strains isolated from patients with cystic fibrosis [15].

### Sphingolipid molecular crystal thin film coating

Sphingolipid solutions were prepared by dissolving either sphingosine (30 mM) or phytosphingosine (30 mM) into organic solvents (i.e., hexane, acetone, or ethanol). Sphingosine or phytosphingosine was added to hexane, which was preheated to 60 °C in a water bath, or ethanol, which was preheated to 70 °C. In some experiments, we used acetone as a solvent, which was preheated to 50 °C. After addition of sphingosine, the solution was agitated and sonicated until the sphingosine aggregates were no longer visible and the solution was clear.

Endotracheal tube segments, vein catheters used as endotracheal tubes in mice and plastic coverslips were used as coating substrates. Endotracheal tube segments were prepared by cutting 1-cm long sections of endotracheal tubes. Sphingolipid films were deposited onto the surface of the aforementioned substrates by dip coating the object into the heated sphingolipid solution prepared as above. The tube segments were manipulated using a 1-mL insulin needle stuck through the plastic and the coverslips were manipulated using straight Kelly forceps. The tube segments/coverslips were immersed in the solution for 1 s, and then slowly withdrawn at a rate of 1 cm/s. Evaporative-induced deposition of the film occurred when the object was exposed to room temperature atmospheric conditions. The resultant films, thus, are not covalent polymers but rather are formed by a rapid crystallization and referred to as molecular crystal thin films. Of note, the plastic coverslips were less resistant to acetone and began to dissolve when dip coated, thus we employed 100% ethanol as the solvent. Ethanol or hexane proved to work equally well and compared to acetone, they are less likely to affect the structural integrity of the PVC tubes. So, we changed our coating process to use ethanol or hexane as our preferred solvent. Repeated dips of up to 10 times were utilized initially, but after coating optimization with ethanol or hexane as solvent, only one dip was necessary to achieve excellent coatings.

Coating with sphingomyelin, ceramide, S1P, and phosphatidylcholine was done according to the above-described method with 30-mM solutions.

In vitro biofilm colonization: complete immersion assay

Endotracheal tube segments were tested for inhibition of bacterial adherence using a modified version of the biofilm colonization model [28, 29]. *P. aeruginosa* and *A. baumannii* were grown overnight on trypticase soy agar (TSA), and *S. aureus* on TSA with 5% sheep’s blood plates at 37 °C. Bacterial suspensions were prepared by placing bacteria into 10-mL trypticase soy broth (TSB) with sterile cotton tip applicators, and incubating on a 120-rpm shaker at 37 °C for 1 h, diluting 1:10 in TSB, measuring absorbance at 550 nm, and diluting with TSB using standard curves prepared for each bacterial strain to achieve 500 colony forming units (CFU)/mL concentration. Sphingolipid-coated, uncoated, or vehicle-coated endotracheal tube segments were immersed in 2-mL bacterial suspension placed in 24-well plates and incubated for 12 h at 37 °C. Endotracheal tube segments were rinsed in 100 mL HEPES/Saline (H/S) (132 mM NaCl, 20 mM HEPES [pH 7.4], 5 mM KCl, 1 mM CaCl_2_, 0.7 mM MgCl_2_, 0.8 mM MgSO_4_) at 37 °C and agitated at 125 rpm for 30 min. Segments were then placed in 10-mL sterile H/S in test tubes and sonicated at 37 °C in a bath sonicator for 10 min to remove adherent bacteria. Test tubes were vortexed for 5 s and the H/S serially diluted, plated on LB plates and incubated overnight. Bacterial CFU were counted and the total number of bacteria adherent to the 1-cm endotracheal tube segments was calculated.

### In vitro biofilm colonization: variation on ISO 22196 and durability testing

Plastic coverslips were tested for the inhibition of bacterial adherence using a modified version of the international standard for measurement of antibacterial activity on plastics and other non-porous surfaces, ISO 22196. Bacteria were prepared as described above to a concentration of 1 × 10^6^ CFU/mL. Ten microliters, i.e., 10,000 CFU, of bacterial suspension was then placed on the sphingolipid- or ethanol-coated portion of the coverslips and covered with a 2 cm × 3 cm low-density polyethylene (LDPE) plastic film and incubated for 24 h at 37 °C. The plastic film was removed and the plastic coverslips rinsed to remove planktonic bacteria. The coverslips were then placed into a drying rack and incubated in humidified air at 37 °C for 12 h. Coverslips were placed into 10-mL sterile H/S in test tubes and sonicated at 37 °C in a bath sonicator for 10 min to remove adherent bacteria. Test tubes were vortexed for 5 s and the H/S serially diluted, plated on LB plates, and incubated overnight. Bacterial CFU were counted and the total amount of bacteria adherent to the coverslips was calculated.

To study the durability of the coating against bacterial adherence we inoculated the coated portion of the coverslip with additional bacteria after 24 and 48 h. Bacteria were prepared the same as for the initial inoculation (1 × 10^6^ CFU/mL). The LDPE plastic film was lifted, 10 μL (10,000 CFU) was pipetted onto the coated surface, and the LDPE film was replaced. The coverslips were incubated again for 24 h and the inoculation was again repeated at 48 h. After 72 h, the coverslips were rinsed in H/S to remove planktonic bacteria, placed in drying racks and incubated for 12 h at 37 °C, sonicated in sterile H/S for 10 min, diluted, plated, and quantification of adherent bacteria was performed.

In order to visualize the adherent bacteria after 24 h, coverslips were removed from the incubator after being rinsed and dried in the humidifier for 12 h. The adherent bacteria were heat-fixed to the coverslips by quickly passing over a Bunsen burner. The coverslips were then immersed in crystal violet for 1 min, serially rinsed in H_2_O, and mounted with VectaMount permanent mounting media. Slides were imaged by light microscopy using a Zeiss Axioimager M.2 microscope.

### In vitro prevention of bacterial biofilm formation

Endotracheal tubes in vivo are exposed to a series of small volume bacterial inoculants in the form of bacteria-containing oral secretions. To investigate the effects of sphingosine and phytosphingosine on biofilm formation on endotracheal tubes mimicking in vivo conditions, tube segments were coated as described above, bacterial suspensions were prepared and endotracheal tube segments were challenged with 10,000 CFU pathogenic bacteria in 10 μL and incubated for 24 h. Adherent bacteria were then released from the surface and quantified.

In addition, 10^5^ CFU *P. aeruginosa* strains 338, 345, or PAO-1 were grown in a 24-well for 48 h, washed 3 times in H/S, resuspended in 200 μL H/S, and adherent bacteria in biofilms were removed from the plate by scraping and by vigorous pipetting. Biofilm formation was confirmed on an additional plate by a spectrophotometric method: Plates were dried for 1 h at 60 °C, stained with crystal violet staining for 5 min, dried for 30 min at 37 °C, washed with H_2_O, and the absorbance was determined at 492 nm using a microplate reader. Bacteria from all wells were combined and 20 μL of the suspension was added to sphingosine-coated endotracheal tubes pieces. The plastic tubes were coated as above in 30 mM sphingosine in ethanol. Samples were incubated at 37 °C for 1 h, bacteria were washed off with 1 mL of H/S and sonication, plated, and CFU were counted after overnight growth.

### In vitro killing assay

Small parts of the tubes were coated with sphingosine or phytosphingosine as above or left uncoated or were treated with C_2_H_5_OH only, placed into wells of a 24-well plate and 10^4^ CFU of *P. aeruginosa*, *A. baumannii*, or *S. aureus* was pipetted as a small drop (5 μl) onto the tube. The samples were incubated for 60 min in a humidified atmosphere, 500 μl TSB were added, and the bacteria were allowed to grow for 1 h. Aliquots of the cultures were then plated and CFU were determined after growth o/n.

### Characterization of sphingolipid coating of endotracheal tubes

Segments of coated endotracheal tubes, as described above, stained with 0.1% osmium tetroxide in H_2_O for 30 min, rinsed in H_2_O for 5 min, dried, cut to fit on standard aluminum specimen mounts, and placed on the mounts using conductive tape. Mounted segments were then sputter-coated with gold/platinum for 15 s and imaged using scanning electron microscopy (SEM) (FEI/Phillips XL-30 SEM). To study the durability of the coating in aqueous solutions samples were immersed in H_2_O, H/S, or PBS for either 12 h or 7 days at 37 °C. Samples were then stained and imaged by SEM.

### Quantification of sphingolipids on endotracheal tubes and in tracheal tissue: mass spectrometry

Sphingosine was extracted from coated plastic surfaces by a one-step lipid extraction. Briefly, the plastic piece was transferred into a siliconized glass tube and sphingosine was extracted by addition of 10 mL methanol and sonication on ice for 1 h. After centrifugation, the lipid extract was diluted with methanol and 50 pmol of C17-sphingosine was added as internal standard. Tissues were processed as previously described [30]. Briefly, samples of murine trachea were homogenized in aqueous-buffered solution on ice using a Bead Ruptor 12 (Omni International, Kennesaw, GA). Aliquots of the homogenates (40 μL), which corresponded to tissue equivalents of 1 mg, were subjected to lipid extraction using 1.5 mL methanol/chloroform (2:1, *v*/*v*). The extraction solvent contained d_7_-sphingosine (Avanti Polar Lipids, Alabaster, AL) as internal standard. Chromatographic separation of sphingosine was achieved by reversed-phase high-performance liquid chromatography (HPLC) (Agilent 1260 series, Agilent Technologies, Waldbronn, Germany) using either an X-Bridge C18 (4.6 × 150 mm, 3.5 μm; Waters, Eschborn, Germany) or a ZORBAX Eclipse Plus C8 (2.1 × 150 mm, 3.5 μm; Agilent) separation column. Water (eluent A) and acetonitrile/methanol (1:1, *v*/*v*; eluent B), both acidified with 0.1% formic acid, were used as eluents. The HPLC column effluent was transferred into an Agilent 6490 triple quadrupole-mass spectrometer via an electrospray ion source interface operating in the positive ion mode (ESI+). The following ion source parameters were used: drying gas temperature 290 °C, drying gas flow 11 L/min of nitrogen, sheath gas temperature 380 °C, sheath gas flow 12 L/min nitrogen, nebulizer pressure 35 psi, capillary voltage 4500 V, and nozzle voltage 2000 V. Ion funnel parameters were: high pressure RF voltage 110 V and low pressure RF voltage 80 V. The following selected reaction monitoring (SRM) transitions were used for quantification (collision energies in parentheses): *m/z* 300.3 → 282.3 (8 eV) for sphingosine, *m/z* 286.3 → 268.3 (8 eV) for C17-sphingosine, and *m/z* 307.3 → 289.3 (8 eV) for d_7_-sphingosine. Quantification was performed with MassHunter Software (Agilent Technologies). Determined sphingolipid amounts were normalized to the actual protein content of the tissue homogenate used for extraction.

### Quantification of sphingosine on endotracheal tubes: sphingosine kinase assay

Sphingosine from pieces of coated endotracheal tubes was extracted in CHCl_3_/CH_3_OH/1 N HCl (100:100:1, *v*/*v*/*v*), the lower phase was dried and resuspended in a detergent solution consisting of 7.5% [*w*/*v*] n-octyl glucopyranoside, 5 mM cardiolipin in 1 mM diethylenetriaminepentaacetic acid [DTPA]. The kinase reaction was initiated by the addition of 0.004 units sphingosine kinase (R&D Systems, Germany) in 50 mM HEPES (pH 7.4), 250 mM NaCl, 30 mM MgCl_2_, 1 μM ATP, and 5 μCi [^32^P]γATP [Hartmann, Cologne, Germany; 3000 Ci/mmol]. Samples were incubated for 60 min at 37 °C with shaking (350 rpm), then extracted in 20 μl 1 N HCl, 800 μl CHCl_3_/CH_3_OH/1 N HCl (100:200:1, *v*/*v*/*v*), 240 μl CHCl_3_, and 2 M KCl. The lower phase was collected, dried, dissolved in 20 μL of CHCl_3_:CH_3_OH (1:1, *v*/*v*), and separated on Silica G60 thin layer chromatography (TLC) plates using CHCl_3_/CH_3_OH/acetic acid/H_2_O (90:90:15:5, *v*/*v*/*v*/*v*) for sphingosine. The TLC plates were analyzed using a phosphorimager. Sphingosine was quantified using a standard curve.

### In vivo infection experiments

*P. aeruginosa* strains 338, 345, or 762 were grown overnight on TSA plates at 37 °C, and *S. aureus* on TSA plates supplemented with 5% sheep blood. Bacteria were removed from the plates, suspended in 40 mL TSA in an Erlenmeyer flask and the density of the bacteria was adjusted to 0.225 OD at 550 nm. Bacteria were then grown for 60 min at 37 °C with 125 rpm. Bacteria were washed twice in H/S and resuspended in H/S. The concentration was determined by measuring the OD at 550 nm using a standard curve and adjusted to 250,000 CFU/μL.

Mice were anesthetized with i.p. xylazine and ketamine, the trachea was exposed, incised and a 21 G plastic catheter was inserted for approximately 1 cm into the trachea. The catheter was coated with sphingosine in hexane or ethanol as described above or, as control, only treated with hexane or ethanol. Approximately 5 mm of the tube protruded from the trachea. The tube was fixed by ligation distal to the insertion site. After a 10-min equilibration, 5 × 10^5^ CFU *P. aeruginosa* strains 338, 345, 762, or multi-resistant *S. aureus* in 2 μl H/S were applied to the outlet of the tube. Bacteria were allowed to infect the lung for 45 min. Mice were then sacrificed, the lung removed under aseptic conditions, homogenized in 3-mL sterile RPMI-1640 buffered with HEPES to pH 7.4, aliquots were plated on LB plates and CFU were determined after overnight growth.

### Sphingosine kinase assays to measure surface sphingosine in tracheal epithelial cells and sphingosine in tracheal mucus

To determine surface sphingosine in tracheal epithelial cells, mice were intubated with ethanol (control)- or sphingosine-coated tubes for 60 min, sacrificed, the intubated part of the trachea immediately removed, opened carefully, and the inner epithelial leaflet was incubated with 0.001 units sphingosine-kinase in 50 mM HEPES (pH 7.4), 250 mM NaCl, 30 mM MgCl_2_, 1 μM ATP, and 5 μCi [^32^P]γATP for 30 min at 30 °C. We ensured that the kinase buffer was only added to the luminal surface of the trachea.

To test whether sphingosine released from the coated tubes remained in the mucus on the tracheal epithelial cells or was also incorporated into the epithelial cells, we isolated trachea as above, but then washed the trachea 5 times in 500 μL H/S to remove the mucus on top of the epithelial cells layer. We then performed the in situ kinase assay as above. Controls were trachea from mice intubated with sphingosine-coated tubes, but the trachea were not washed and immediately used for the in situ sphingosine kinase assay.

The kinase reaction was terminated by placing the trachea in 100 μl H_2_O, followed by addition of 20 μL 1 N HCl, 800 μl CHCl_3_/CH_3_OH/1 N HCl (100:200:1, *v*/*v*/*v*), 240 μL CHCl_3_, and 2 M KCl. Phases were separated, the lower phase was dried, dissolved in 20 μL of CHCl_3_:CH_3_OH (1:1, *v*/*v*) and separated on Silica G60 thin layer chromatography (TLC) plates using CHCl_3_/CH_3_OH/acetic acid/H_2_O (90:90:15:5, *v*/*v*/*v*/*v*) to develop the plates. The TLC plates were analyzed on a Fuji-phosphorimager and sphingosine amounts were calculated using a standard curve.

In addition, we also determined sphingosine in the wash buffer of the trachea. To this end, a 200-μl aliquot of the buffer was extracted with CHCl_3_/CH_3_OH/HCl (100:100:1; *v*/*v*/*v*), the organic phase was dried, resuspended in 20 μL detergent solution, sonicated for 10 min, and 100 μl of the sphingosine kinase buffer were added. The samples were incubated for 60 min at 37 °C, 20 μL 1 N HCl, 800 μL CHCl_3_/CH_3_OH/1 N HCl (100:200:1, *v*/*v*/*v*), each 240 μl CHCl_3_ and 2 M KCl were added and the samples were further processed as above.

### Sphingosine immunostainings to determine sphingosine in tracheal epithelial cells

Mice were anesthetized and intubated with sphingosine-coated or ethanol-, i.e., control-coated tubes for 60 min as above. Additional controls were left untreated. Mice were sacrificed, the intubated part of the trachea or the trachea of the untreated controls were removed and immediately fixed with 4% PBS-buffered paraformaldehyde (PFA, Roth, Karlsruhe, Germany) for 36 h. The tissue was serially dehydrated with an ethanol to xylol gradient and then embedded in paraffin. Trachea were sectioned at 7 μm, dewaxed, rehydrated, and treated with pepsin (Digest All; #003009, Invitrogen, Carlsbad, USA) for 30 min at 37 °C, washed each once with water and PBS and blocked for 10 min at room temperature with PBS, 0.05% Tween 20 (Sigma), and 5% fetal calf serum (FCS). The samples were then incubated with anti-sphingosine antibodies (1:1000 dilution; Alfresa Pharma Corporation, Japan) in H/S supplemented with 1% FCS at room temperature for 45 min. Samples were washed three times with PBS plus 0.05% Tween 20 and once with PBS. The tissue was secondarily labeled with Cy3-coupled anti-mouse IgM F(ab)_2_ fragments (Jackson Immunoresearch, West Grove, USA) in H/S, 1% FCS for 45 min. The samples were again washed 3-times with PBS plus 0.05% Tween 20 and once with PBS and embedded in Mowiol. Samples were evaluated by confocal microscopy on a Leica TCS-SP5 confocal microscope using a × 40 lens. All samples were measured at identical settings. Images were analyzed with Leica LCS software version 2.61 (Leica Microsystems, Mannheim, Germany). All data were quantified using Image J and are expressed as arbitrary units (a.u.). We analyzed trachea from 6 mice per experimental group. In each sample, the fluorescence of 100 randomly chosen different tracheal epithelial cells was quantified. The control stainings with only Cy3-coupled isotype control antibodies were very weak.

### Total sphingosine in the trachea after intubation

To determine total sphingosine in trachea after intubation, mice were intubated for 60 min with ethanol-treated or sphingosine-coated tubes as above, the tube removed, the trachea isolated, and shock frozen. Sphingosine was quantified by mass spectrometry as above.

### In vivo stability experiments

To test the in vivo stability of sphingosine coated onto endotracheal tubes, 21 G tubes were coated with sphingosine in hexane or ethanol as described above or, as control, only treated with hexane or ethanol. Tubes were then inserted into the trachea of mice for 60 min, removed, and stored for 24 h at room temperature. This procedure was repeated 4 times over 4 days. We employed this repeated exposure, because it is difficult to obtain a stable anesthesia of mice over 4 h and, in addition, we also obtained information on stability of the coating over time. Controls were hexane- or ethanol-coated tubes that were also inserted and sphingosine-, hexane or ethanol-coated tubes that were not inserted and coated either together with the inserted tubes and stored for 4 days or tubes that were coated immediately prior to quantification of surface sphingosine. Sphingosine on the surface (inner and outer surfaces) was quantified by a sphingosine kinase assay as described above.

### In vivo toxicity of and induction of inflammation by sphingosine-coated catheters

PVC tubes were coated with 30 mM sphingosine as above and mice were intubated for 2 h as above. The trachea was removed, fixed with 4% PFA, embedded in paraffin, sectioned at 6 μm, and stained with H&E and TUNEL (TMR-Red kit) according to the instructions of the vendor (Roche, Mannheim). To prove the function of the TUNEL, trachea sections were treated with DNAse for 30 min at 37 °C, which resulted in 100% TUNEL positive cells. In addition, paraffin sections were stained with Cy3-labeled anti-CD45 antibodies to detect any influx of leukocytes. To this end, the samples were dewaxed, blocked for 10 min in 5% fetal calf serum (FCS) in H/S, washed in PBS, stained for 45 min with anti-Gr1 antibodies (1:250 diluted in H/S + 1% FCS, BD, Heidelberg, Germany), washed three times in PBS supplemented with 0.05% Tween 20, incubated with Cy3-labeled anti-rat IgG antibodies for 45 min (1:500 in HS/1% FCS, Jackson-ImmunoResearch), washed again 3 times in PBS/0.05% Tween 20 and once in PBS, and embedded in Mowiol. As positive controls, i.e., to verify the anti-CD45 antibody staining, we used spleen sections. All samples were analyzed on a Leica TCS SL confocal microscopy.

The number of dead epithelial cells and the influx of leukocytes were quantified in 10 sections from each trachea. We analyzed 6 mice, i.e. a total of 60 sections. In each section, the entire epithelial cell layer and the submucosa of the trachea were investigated. Image J was used for analysis of the samples.

### Quantification and statistical analysis

Data are expressed as arithmetic means ± SD. For the comparison of continuous variables from independent groups, we used one-way ANOVA followed by post hoc Student’s *t* tests for all pairwise comparisons, applying the Bonferroni correction for multiple testing. The *P* values for the pairwise comparisons were calculated after Bonferroni correction. All values were normally distributed and the variances were similar. A *p* value of 0.05 or less (two-tailed) was considered statistically significant. The sample size planning was based on the results of two-sided Wilcoxon-Mann-Whitney tests (free software: G*Power, Version 3.1.7, University of Duesseldorf, Germany). Investigators were blinded for histology experiments.
